# Nucleotide substitutions in dengue virus serotypes from Asian and American countries: insights into intracodon recombination and purifying selection

**DOI:** 10.1186/1471-2180-13-37

**Published:** 2013-02-14

**Authors:** Susanta K Behura, David W Severson

**Affiliations:** 1 Eck Institute for Global Health, Department of Biological Sciences, University of Notre Dame, 46556, Notre Dame, IN, USA

**Keywords:** Dengue virus, Nucleotide substitution, Codon usage, Serotype, Intracodon recombination, Purifying selection, Fixed mutations

## Abstract

**Background:**

Dengue virus (DENV) infection represents a significant public health problem in many subtropical and tropical countries. Although genetically closely related, the four serotypes of DENV differ in antigenicity for which cross protection among serotypes is limited. It is also believed that both multi-serotype infection as well as the evolution of viral antigenicity may have confounding effects in increased dengue epidemics. Numerous studies have been performed that investigated genetic diversity of DENV, but the precise mechanism(s) of dengue virus evolution are not well understood.

**Results:**

We investigated genome-wide genetic diversity and nucleotide substitution patterns in the four serotypes among samples collected from different countries in Asia and Central and South America and sequenced as part of the Genome Sequencing Center for Infectious Diseases at the Broad Institute. We applied bioinformatics, statistical and coalescent simulation methods to investigate diversity of codon sequences of DENV samples representing the four serotypes. We show that fixation of nucleotide substitutions is more prominent among the inter-continental isolates (Asian and American) of serotypes 1, 2 and 3 compared to serotype 4 isolates (South and Central America) and are distributed in a non-random manner among the genes encoded by the virus. Nearly one third of the negatively selected sites are associated with fixed mutation sites within serotypes. Our results further show that of all the sites showing evidence of recombination, the majority (~84%) correspond to sites under purifying selection in the four serotypes. The analysis further shows that genetic recombination occurs within specific codons, *albeit* with low frequency (< 5% of all recombination sites) throughout the DENV genome of the four serotypes and reveals significant enrichment (p < 0.05) among sites under purifying selection in the virus.

**Conclusion:**

The study provides the first evidence for intracodon recombination in DENV and suggests that within codons, genetic recombination has a significant role in maintaining extensive purifying selection of DENV in natural populations. Our study also suggests that fixation of beneficial mutations may lead to virus evolution via translational selection of specific sites in the DENV genome.

## Background

Dengue virus (DENV) infection causes dengue fever, dengue shock syndrome and dengue hemorrhagic fever in humans. According to the new guidelines released by World Health Organization in 2009, these diseases are now classified as dengue, dengue with warning signs and severe dengue. The virus is primarily transmitted by *Aedes aegypti* mosquitoes. DENV poses a significant public health threat in many subtropical and tropical countries. More than 500,000 dengue infected patients, including large numbers of children, are hospitalized each year in more than 100 countries [1]. Many of them (>20,000) die due to complications arising from the infection.

The DENV genome (~ 11 kb) is composed of a positive-sense single-stranded RNA. The genome encodes three structural proteins: capsid (C), pre-membrane/membrane (prM/M), and envelope (E), and seven non-structural (NS) proteins: NS1, NS2A, NS2B, NS3, NS4A, NS4B, and NS5, flanked by 5^′^- and 3^′^-non-translated regions (5^′^-NTR/3^′^-NTRs). A single open reading frame (ORF) in the genome is used to synthesize a polypeptide of ~ 3400 amino acids which is then post-translationally cleaved to produce the individual proteins.

There are four serotypes (DENV-1, DENV-2, DENV-3 and DENV-4) of dengue virus. Although genetically closely related, the dengue serotypes differ in antigenicity for which cross protection among serotypes is limited [2,3]. Disease severity of dengue is often attributed to secondary infection with a virus belonging to a serotype other than that of the primary infection, but evolution of the virus is also considered as a significant contributing factor to increased epidemics of dengue [4]. It is also believed that both multi-serotype infection as well as the evolution of viral antigenicity may have confounding effects in increased dengue epidemics [5]. Numerous studies have been performed that investigated genetic diversity of DENV, both in time and space as reviewed in [6,7], but the precise mechanism(s) by which dengue viruses cause severe haemorrhagic disease is not well understood [8].

Understanding molecular patterns and selection features associated with natural populations of DENV serotypes has provided useful clues to study dengue epidemiology [9-12]. The study by Zanotto *et al*., 1996 [13] revealed that the most common pressure acting on DENV in nature is purifying selection, the form of natural selection that removes deleterious mutations often referred to as negative selection. On the other hand, positive selection increases the frequency of mutations that confer a fitness advantage to individuals carrying the alleles. Adaptive evolution results from propagation of advantageous mutations in the population which is largely driven by positive selection. A number of amino acid positions were identified within the envelope (E) glycoprotein that have been subject to relatively weak positive selection in both DENV-3 and DENV-4, as well as in two of the five “genotypes” of DENV-2 [14-16]. These studies suggested adaptive evolution of DENV in natural populations but indicated that the adaptive selection pressures differ among serotypes, genotypes and the encoded proteins of the virus. Furthermore, fixation of beneficial mutations may lead to virus evolution with altered antigenicity, virulence, or tissue tropism; and eventually influence disease patterns and transmission [17]. Similarly, genetic recombination is also a significant factor in diversity of DENV in natural populations [18]. However, no information is available indicating whether recombination within codons plays a role in natural selection of DENV. Recent studies show that intracodon recombination is more prominent in highly evolving organisms including viruses and bacteria [19,20]. Intracodon recombination is the form of genetic recombination wherein nucleotide triplets of the same codon undergo sequence exchange via breakpoints within the codon. The mechanisms of evolutionary processes that produce such events are described elsewhere [20]. Based on coalescent simulation of codon sequences, it has been shown [20] that intracodon recombination does not have a strong overall effect on the generation of non-synonymous changes but significantly affects synonymous changes.

In the present study, we investigated genetic diversity and nucleotide substitution patterns in each of the four serotypes of DENV represented in samples from Asian and South and Central American countries that were sequenced as part of the ‘Genome Resources in Dengue’ (GRID) project at the Broad Institute. The primary objectives of our study were to 1) assess substitution patterns in DENV genome coding regions, 2) determine if synonymous substitution sites were linked with translational selection of genes, 3) identify selection sites and nature of selection, and 4) test associations between selection and recombination in DENV serotypes. The results obtained from this study provide insights into the nature of mutational patterns in DENV in a genome-wide manner and reveal evidence for translational selection (selection associated with increased efficiency and accuracy of translation of genes to proteins) of specific sites between Asian and American DENV genomes. The results from this study also provide the first evidence for intracodon recombination and its association with purifying selection in each serotype.

## Methods

### Dengue virus, genetic and phylogenetic analysis

The current study was performed with whole genome sequences of dengue virus representing the four serotypes. A total of 260 genome sequences were included in the study. The sample collection and generation of sequence data was carried out by the GRID project. The sequence data is publicly available to the research community at http://www.broadinstitute.org/annotation/viral/Dengue/Home.html. We randomly sampled equal numbers (n = 65) of whole genome sequences for each serotype for the current investigation. The accession number for the individual DENV genome sequences, country of origin and year of collection for each sample used in this study is provided in Additional file 1. The sample information is based on data provided by the GRID project. The DENV genome sequences analyzed in the current study represent serotypes 1, 2 and 3 from multiple countries of Asia and Central and South America, whereas samples of serotype 4 were collected from either Central or South American countries. That is, only 68 genome sequences of serotype 4, all representing collections from the Americas (none from Asia) were available in the GRID project database at the time of this investigation.

The codon-based sequence alignments of the genome sequences of each serotype were generated by ClustalW [21] and inspected by eye to confirm correct alignment of start and end codons for all sequences. The sequences were aligned within serotypes. The phylogenetic relationships among sequences were inferred using the Neighbor-Joining method implemented in MEGA4 [22]. The evolutionary distances were computed using the Kimura-2 method and are reported as the number of nucleotide substitutions per site. The nucleotide diversity per site was determined by DnaSP software [23]. The average number of amino acid substitutions per site, number of haplotypes within each serotype, and population mutation rate among samples within serotype were determined from MEGA4 and DnaSP software.

### Analysis of synonymous and non-synonymous mutations

The synonymous and non-synonymous sites were detected by DnaSP software. The number of nucleotide changes at each site of the codon position was compared with the positions of synonymous and non-synonymous sites to determine which codon position contributed to change of amino acid sequence and also change from one codon to an alternate synonymous codon. Fixation of mutations was inferred from the allele frequencies of each mutation between the two groups within serotype defined by the phylogenetic analysis. For serotype 1, 2 and 3, the Asian and American DENV samples represented two distinct populations phylogenetically. For serotype 4, the Central and South American samples were classified as distinct phylogenetic groups. If a mutation had one allele with frequency >95% in one group and frequency ≤ 5% in the other group, the mutation was considered ‘fixed’ in the serotype.

### Identification of selection sites

The “fixed effects likelihood (FEL)” method [24] was used for this purpose. The method relies upon fitting two models (one for nucleotide sequences and another for codon sequences) by likelihood methods to estimate the number of non-synonymous (dN) and synonymous (dS) changes for each site. Then based on the two model parameters α (instantaneous synonymous site rate) and β (instantaneous non-synonymous site rate), likelihood ratio tests are conducted to infer statistical significance of higher dN over dS (positive selection) or *vice versa* (negative selection or purifying selection) of the sites.

### Codon bias analysis

We wanted to know how nucleotide substitutions affect codon usages in the samples. First, the relative synonymous codon usage (RSCU) indices were calculated for individual codons within each serotype. These indices show if specific codons are used more often or less often in the observed sequence data than expected. The expected value of codon usage is calculated as the ratio of total number of amino acid counts divided by the number of synonymous codons that code for the amino acid. Then the RSCU values are calculated as the ratio of the observed number of codons to the expected number. The stop codons were included for this analysis. Also, Trp and Met codons were excluded from this analysis as only one codon is used to code for these amino acids. The preferred and non-preferred codons have RSCU > 1 and RSCU < 1, respectively. Based on this, each synonymous substitution site was examined to determine whether it corresponded to a preferred codon or non-preferred codon. The codon context analysis was performed using the Anaconda software [25,26]. It includes a set of statistical and visualization methods to reveal information about codon context (sequential patterns of codons in a gene), codon usage bias as well as nucleotide repeats within open reading frames (ORFeome). We used the cluster analysis tool, which is based on calculating similarities between two vectors of the contingency tables of codon frequencies, to group codon pairs (represented by rows and columns of the correlation matrix of residual values for each serotype). The cluster patterns represented global patterns of codon contexts within each serotype.

### Analysis of recombination

Population recombination analyses in DENV were performed using the composite likelihood method of Hudson 2001 [27], but adapted to finite-sites models (applicable to diverse genomes such as those of some viruses and bacteria) [28]. The PAIRWISE program included in the LDhat package (freely available at http://ldhat.sourceforge.net/), a suite of population genetic recombination tools [28] was implemented to analyze recombination in each serotype of DENV. The PAIRWISE program performs estimation of the population-scaled recombination, 2Ner for haploid species, where Ne is the effective population size and r is the genetic map distance across the region. The composite likelihood method implements a finite-sites model to estimate the coalescent likelihood of two-locus haplotype configurations. The coding sequences of DENV genomes within each serotype were formatted by ‘Convert’, a program included in LDhat, to generate data files of sites and positions of mutations in the sequences of the sample. Then these files were used in the PAIRWISE analysis to generate likelihood lookup tables for sequence data of each serotype. The likelihood values utilized the estimated Watterson’s theta per site, 100 as the maximum value of 2Ner for the grid and 101 as the number of points on the grid as recommended. The minimum numbers of recombination events (Rmin) were estimated by the Hudson and Kaplan (1985) [29] method to describe the evidence for recombination across the coding region of the DENV genome. The dynamic programming algorithm of Myers and Griffiths (2003) [30] implemented in the PAIRWISE program was used to identify a list of all pairs of sites with evidence of recombination. The positions of these pairs of sites in the DENV genome were used to determine if they are localized within codons (intracodon).

### Coalescent simulation of codon sequences

The codon sequences of dengue virus serotypes were simulated by the coalescent method of Arenas and Posada (2010) [20]. It is based on the coalescent with recombination method under a Wright-Fisher neutral model [31]. The ‘Netcodon’ algorithm developed by Arenas and Posada (2010) [20] was used to simulate DENV codon sequences with serotype specific recombination rates estimated by PAIRWISE and the M1 codon model. This codon model incorporates two categories (ω0 P0, ω1 P1) of values to represent proportions (P0 or P1) of non-synonymous to synonymous substitutions (ω0 or ω1) in the sample sequences. The other parameters such as mutation rate, nucleotide frequency of coding sequences, transition/transversion ratio estimated from the observed data by DnaSP [23] were used in generating simulated data sequences. The simulation was carried out to generate 10 replicates of 65 samples, which generated 650 random sequences of the DENV coding genome. The simulated data were then analyzed by PAIRWISE to identify all the pair-wise sites showing evidence of recombination and to determine if they are localized within codons (intracodon).

### Statistical analysis

All statistical analyses were performed in R. The 2x2 contingency tests were conducted either by Yeats’s Chi square tests or by Fisher’s Exact tests depending upon the sample sizes. All p-values are two-tailed. Statistical significance of association between intracodon recombination and purifying selection was measured by hypergeometric tests as per method described in Fury *et al*. (2006) [32]. Briefly, the distribution of sites of purifying selection (n1) and the sites showing intracodon recombination (n2) among all the recombination sites (n, which are identified from PAIRWISE analysis) were determined. The total number of possible choices for the two groups of sites was calculated as C(n, n1)* C(n, n2). Similarly, the total number of possibilities for choosing the purifying sites was C(n, n1), whereas the number of possibilities for choosing the purifying sites showing evidence of intracodon recombination was C(n1, m), where m is the total counts of sites showing evidence of both purifying selection and recombination within codons. Among the total number of sites in the genome identified as sites with intracodon recombination, the remaining n2-m sites were chosen among the remaining n-n1 purifying sites in C(n − n1, n2 − m) ways. Thus, the probability of hypergeometric distribution of sites showing association between the two (purifying selection and intracodon recombination) was calculated as [C(n, n1) × C(n1, m) × C(n − n1, n2 − m)] / {C(n, n1)* C(n, n2)}. P-values < 0.05 were considered statistically significant unless stated otherwise.

## Results

### Dengue virus serotypes and genetic diversity

**Figure 1:**
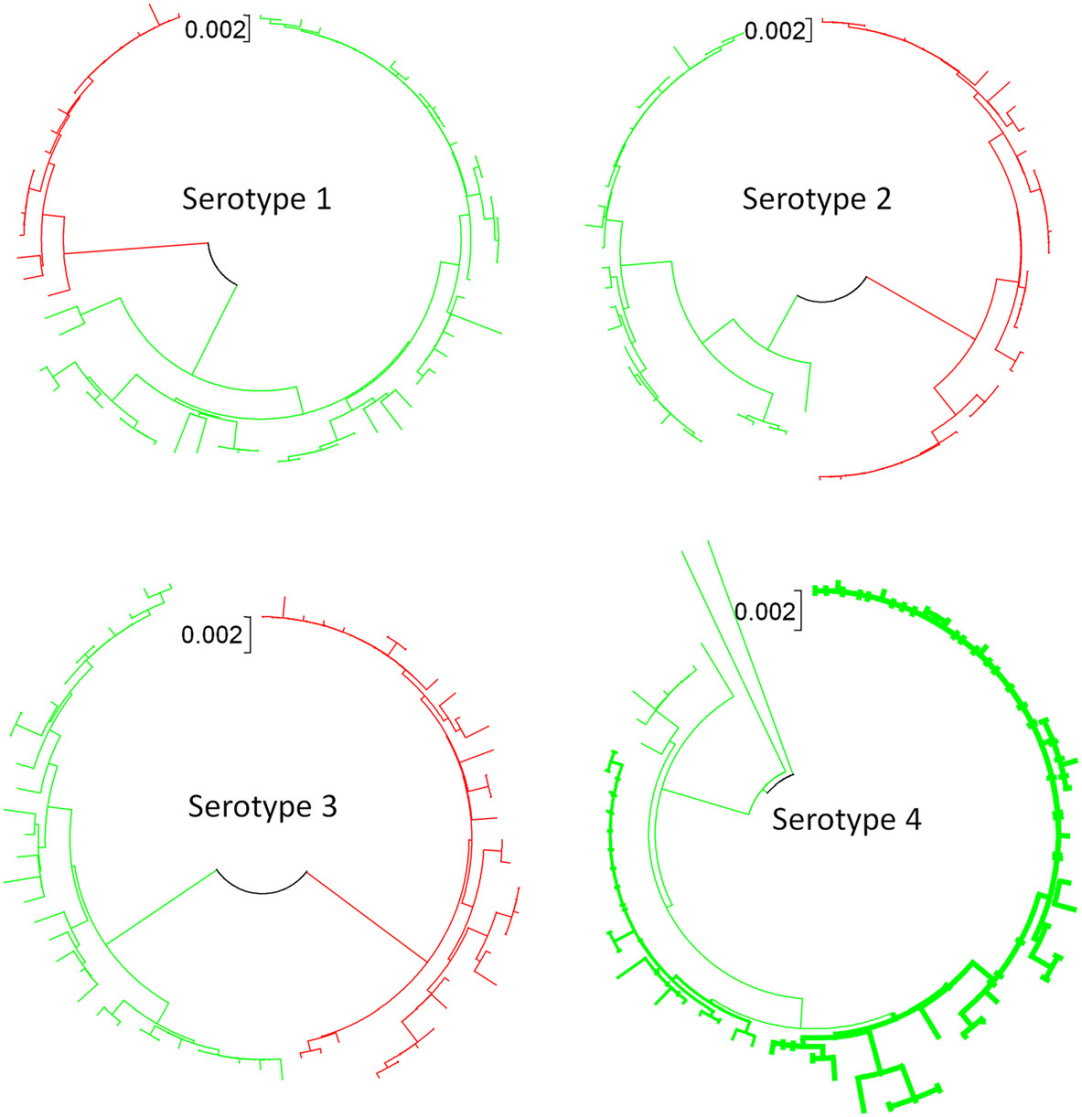
**Geographical structuring within dengue virus serotypes evident from phylogenetic (neighbor-joining tree) analysis.** Asian isolates (red) and American isolates (green) are compared for serotypes 1, 2 and 3. For serotype 4, isolates from Central America (light green) are compared with isolates from South America (dark green). The unit of branch length is shown for each tree.

The sequence data investigated in this study represent genome-wide coding sequences of DENV (n = 260 isolates) from different countries. While samples of DENV serotype 1, 2 and 3 are derived from both Asian and American countries, the collections of serotype 4 are limited to Central and South American countries (Additional file 1). The sequences of serotype 4 available by the GRID project are only from Americas. Thus, serotype 1, 2 and 3 sequences represented geographically more diverse samples unlike the serotype 4 sequences. Accordingly, the genetic diversity observed within serotype 1, 2 or 3 samples was higher than that of serotype 4 samples. The average number of nucleotide differences ranges from 168 to 492 among the samples. The nucleotide diversity (π) is ~ 0.04 among samples belonging to serotype 1, 2 and 3 and 0.01 for serotype 4. The neighbor-joining phylogenetic tree analyses of the coding sequences also show that samples of serotype 1, 2 and 3 are associated with two groups corresponding to Asian and American DENV isolates whereas those of serotype 4 represent a monophyletic group (Figure 1). However, diversity within serotype 4 is also evident that corresponds to the Central and South American DENV isolates, respectively. More than 80% of the nucleotides in the coding sequences of the DENV genome remain fixed. Although this suggests that these isolates are genetically very similar, about 1500 to 2000 sites (15% - 18% of the total sites) reflect nucleotide substitutions among them across serotypes. Furthermore, the relative rate of transition versus transversion substitutions (Additional file 2) also suggests that the nucleotide substitution patterns are biased towards excess transitions over transversions among the samples in each serotype.

### Synonymous and non-synonymous substitutions

**Figure 2:**
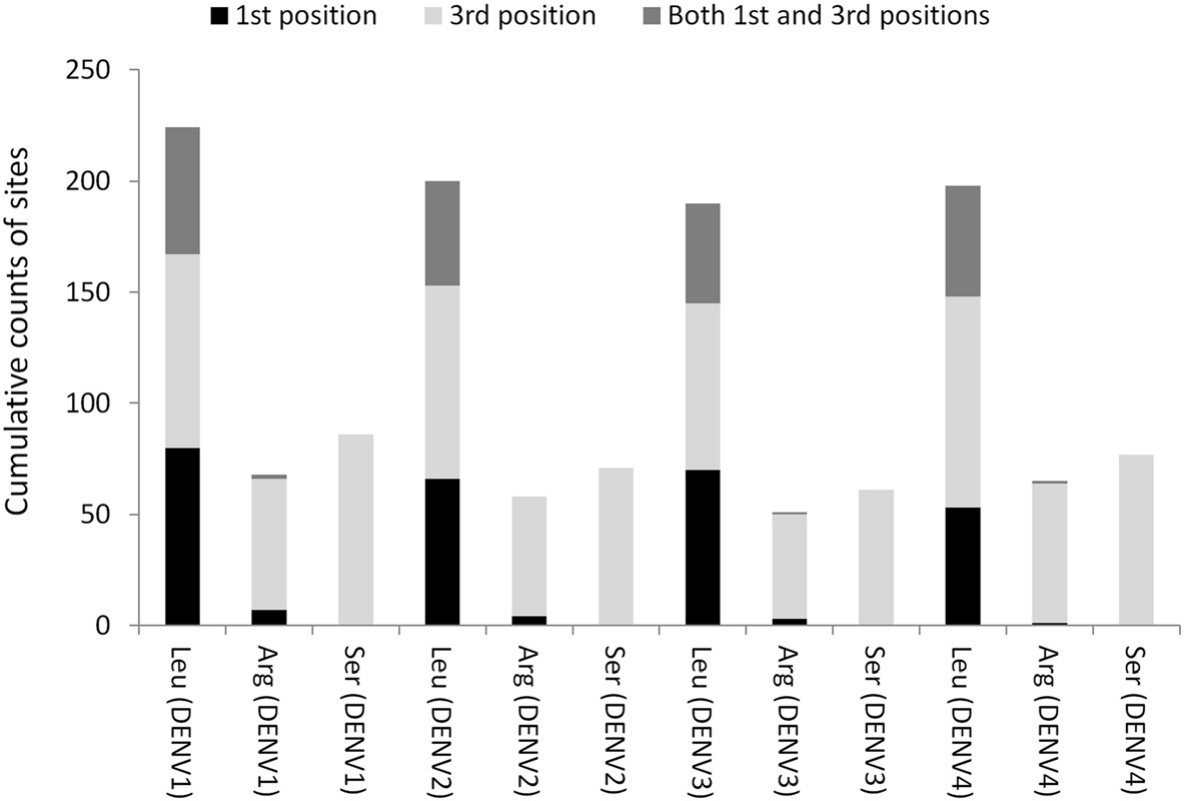
**Distribution of substitution sites in codons.** Stacked bar graphs show the distribution of substitution sites in the 1^st^, 3^rd^ and 1^st^ + 3^rd^ positions of specific codons in dengue virus serotypes.

The counts of synonymous and non-synonymous substitution sites are shown in Table 1, and indicate that nearly 80% of all the substitutions in the DENV genome are synonymous. The number of synonymous and non-synonymous changes at 1^st^, 2^nd^ and 3^rd^ codon positions of each serotype is also shown in Table 1. It shows that the number of silent changes at the 1^st^ position of codons among the samples of serotypes 1, 2 and 3 are similar to that of serotype 4, in spite of differences in the overall nucleotide diversity among the serotypes. However, as expected, most of these changes are associated with the 3^rd^ position of codons. The results further reveal that many codons for Leu, Ser and Arg are associated with more than one substitution in the same codon. The Leu codons are associated with nucleotide substitutions at either the 1^st^ or 3^rd^ position or at both 1^st^ and 3^rd^ positions with nearly similar proportions (Figure 2). Figure 2 clearly shows that a similar pattern is absent in the Arg and Ser codons. The silent changes of Arg and Ser codons are mostly in the 3^rd^ position, although changes in the 1^st^ position are also evident. This suggests that 1^st^ positions in DENV Ser and Arg codons, but not the Leu codons may be under selection (translational) constraint. There are no changes at the 2^nd^ position of codons in dengue virus isolates we examined (although serine codons can have such silent changes).

**Table 1:** Number of synonymous and non-synonymous changes in DENV serotypes

**Category**	**Position 1**	**Position 2**	**Position 3**	**Codons**
**DENV1-Syn**	152	0	1333	1420
**DENV1-Nonsyn**	128	112	129	244
**DENV2-Syn**	120	0	1212	1281
**DENV2-Nonsyn**	109	96	111	211
**DENV3-Syn**	121	0	1129	1197
**DENV3-Nonsyn**	102	117	100	218
**DENV4-Syn**	112	0	1259	1370
**DENV4-NonSyn**	102	103	109	314

Dengue virus serotypes are listed as DENV1, DENV2, DENV3 and DENV4. Syn: synonymous changes. Nonsyn: non-synonymous changes. Position 1/2/3: 1^st^, 2^nd^ and 3^rd^ positions of codons. For synonymous changes, the 3^rd^ position substitutions are predominant as expected. However, for non-synonymous changes, all the three positions of codons undergo changes with no significant bias with any specific position. Number of codons associated with non-synonymous (Non-syn) or synonymous (Syn) changes in each serotype are shown in the last column.

We observed that the non-synonymous substitutions (~ 300 in total) are distributed in nearly equal numbers among the three codon positions (Table 1). Although 1^st^ and 2^nd^ codon positions are generally associated with non-synonymous changes of codons, this result suggests that there is no such bias of specific codon positions in accumulating non-synonymous changes in DENV. It was further found that, in the DENV genome, synonymous and non-synonymous changes occur at more than one position (1^st^, 2^nd^ and 3^rd^ positions of codons) within codons (Table 2). Of note, while substitutions at multiple positions within non-synonymous codons are as frequent as single substitutions with isolates of serotypes 1, 2 and 3, substitutions at multiple positions were absent among the serotype 4 isolates. The non-synonymous changes account for an average of 0.013 to 0.018 amino acid substitutions per site in serotypes 1, 2 and 3, and 0.005 in serotype 4.

**Table 2:** Synonymous and non-synonymous changes at one or more positions within codons

**Serotype**	**Nonsyn_multiple**	**Nonsyn_single**	**Syn_multiple**	**Syn_single**
DENV-1	125	119	85	1355
DENV-2	105	106	55	1231
DENV-3	101	117	45	1148
DENV-4	0	314	58	1312

More than one codon position is associated with synonymous (Syn) as well as non-synonymous (Nonsyn) changes in DENV. The numbers of such codons (Syn_multiple and Nonsyn_multiple) are shown along with codons where only one site has a substitution.

### Fixation of synonymous *versus* non-synonymous substitutions in DENV genes

Many substitutions show fixation tendency in DENV. This was more prominent among the inter-continental isolates (Asian and American) of serotypes 1, 2 and 3 compared to serotype 4 isolates (South and Central America). The number of such sites and the total number of substitutions in individual genes are listed in Additional file 3. It shows that the fixed sites are differentially distributed among the genes. Based on 2x2 contingency tests (Pearson Chi Square), it was found that synonymous or non-synonymous sites which show fixation or non-fixation tendency among samples are significantly biased in specific genes of DENV. Genes encoding membrane glycoprotein precursor M, envelope protein E, and nonstructural proteins NS2A and NS5 show significant bias in the fixed and non-fixed substitutions in serotype 1. In serotype 2, only two genes (anchored capsid protein C and nonstructural protein NS4B) show such a pattern, whereas only one gene (nonstructural protein NS3) reflects this pattern in serotype 3. In the serotype 4 isolates, no gene shows such substitution sites. In serotype 4 isolates, only 2-4% of the substitution sites are fixed between Central and Southern American DENV isolates compared to 30-40% of such sites between Asian and American serotype 1, 2 and 3 DENV isolates.

It was also observed that geographical populations within serotypes show extensive codon usage bias. Based on the relative synonymous codon usage (RSCU) index of dengue samples of Asia and America (or South and Central America), it was found that codon preferences or non-preferences were significant between geographical origins (Table 3)**.** In the serotype 3 isolates, the association of codon preferences or non-preferences between American and Asian countries was not significant, although rare codon fixation was higher in frequency than that of frequent codons.

**Table 3:** Codon usage bias in dengue virus

**DENV-1**	**RSCU > 1 in Asian DENV**	**RSCU < 1 in Asian DENV**	**p value**
**RSCU > 1 in American DENV**	91	160	0.02
**RSCU <1 in American DENV**	193	112	
**DENV-2**	RSCU > 1 in Asian DENV	RSCU < 1 in Asian DENV	
**RSCU > 1 in American DENV**	35	121	0.000004
**RSCU <1 in American DENV**	155	79	
**DENV-3**	RSCU > 1 in Asian DENV	RSCU < 1 in Asian DENV	
**RSCU > 1 in American DENV**	80	132	0.08
**RSCU <1 in American DENV**	138	116	
**DENV-4**	RSCU > 1 in Central American DENV	RSCU <1 in South American DENV	
**RSCU > 1 in Central American DENV**	5	13	0.004
**RSCU <1 in South** **American DENV**	22	16	

Number of synonymous codons that are used either more than expected (frequent) or less than expected (rare) between Asian and American isolates of DENV serotype 1, 2 and 3 (or between South and Central American isolates of DENV4).

Interestingly, the most biased codon usage (at least two fold change in RSCU) is associated with codons of four amino acids: Gly, Pro, Ser and Thr (Additional file 4). These amino acids are among the abundant residues in DENV proteins (each contributes to >4% of total amino acid residues; note that the percentage of representation of the 20 amino acids to DENV proteins ranges from 1 to 10). The number of sites that are preferred in DENV is relatively less in number than the sites that are associated with non-preferred codons, a pattern which is consistent irrespective of geographical origin. This suggests that the balance between mutation and codon selection in dengue virus is probably maintained irrespective of geographical structuring within serotypes.

### Context patterns of nucleotides in coding sequences

**Figure 3:**
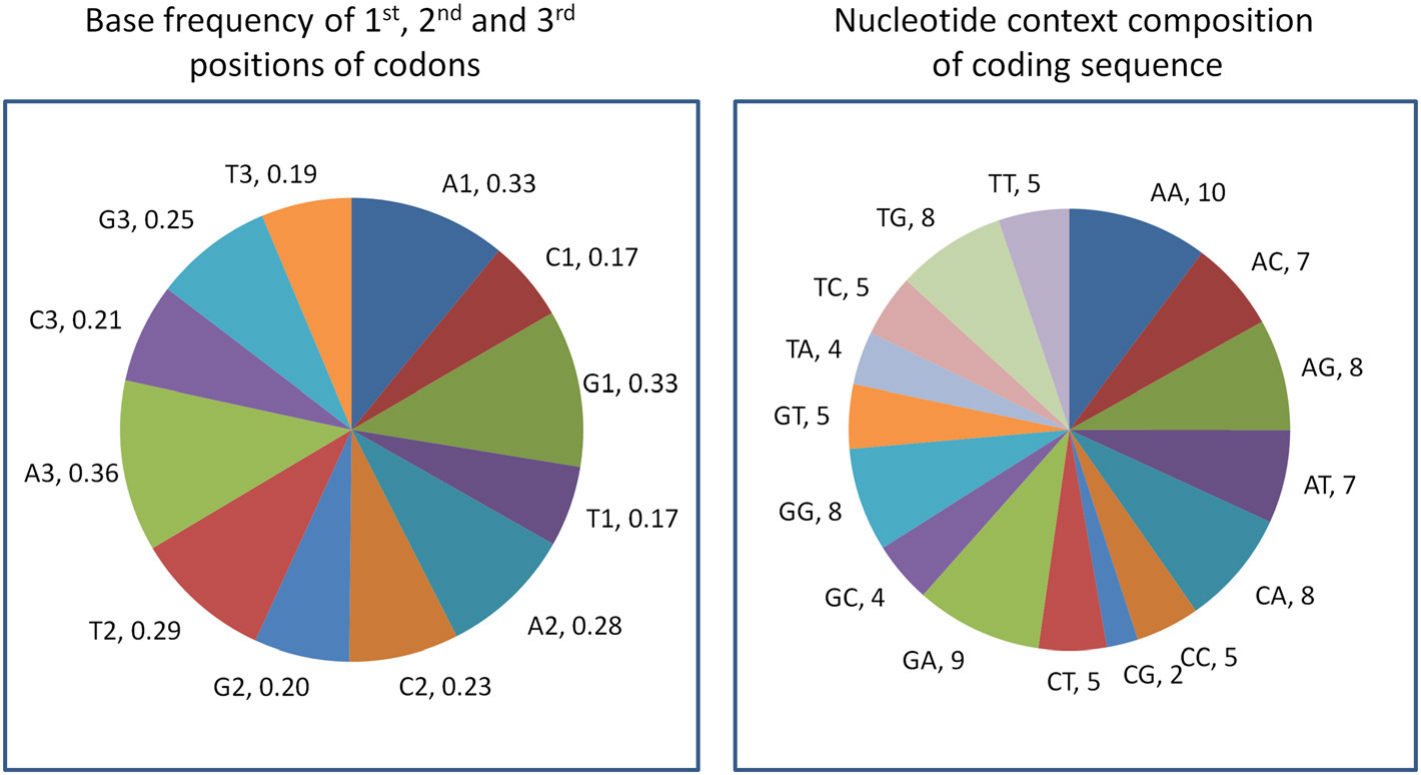
**Distribution of nucleotide frequency in codons.** Pie chart representation of mean frequencies of the four nucleotides at 1^st^, 2^nd^ and 3^rd^ positions of codons in dengue virus (left). The chart on the right shows nucleotide context pattern (based on mean dinucleotide frequencies) in the coding sequences of dengue virus. The number after each nucleotide and nucleotide pair represents its proportion compared to the total nucleotide counts for that codon position (left) or total counts of dinucleotides in the coding sequences (right). The nucleotide frequency as well as the dinucleotide frequency varies in highly correlated manner (Pearson correlation > 0.93), hence the mean value represents the nucleotide composition pattern of coding sequences of DENV within as well as between the four serotypes.

The nucleotide context patterns of codon sequences of DENV were investigated. The base frequencies of 1^st^, 2^nd^ and 3^rd^ positions of codons are shown in Figure 3. It shows that A and G frequencies are relatively higher than C and T in the 1^st^ positions of codons, whereas frequencies of A and T are relatively more frequent than that of C and G in the 2^nd^ positions of codons in all four serotypes. On the other hand, in the 3^rd^ positions of codons, the frequency of A is higher than that of C, G or T. The 3^rd^ position of codons, being the silent position, this result suggests that A-ending codons are preferred in DENV genes. This pattern is highly consistent among the samples in each serotype (data not shown). The nucleotide context patterns (i.e., given a nucleotide, how frequently it makes neighboring context with itself or the other three nucleotides) were also investigated in the coding sequences of the samples. Figure 3 shows frequency of each of the 16 possible nucleotide contexts. It shows that AA and GA nucleotide contexts are relatively more frequent than any other contexts in the coding sequences of the DENV genome. The CG contexts are least abundant in DENV genes. This pattern of nucleotide context frequencies is very similar among the samples in each serotype (Pearson correlation coefficient is greater than 0.93).

The codon context maps of DENV genomes for the four serotypes were generated using the Anaconda algorithm [26]. The codon context maps for each serotype show the relative propensity of each codon to pair with either itself or other codons (61x61 possible pairs) (Additional file 5). The maps indicate that although codon context patterns are overall highly similar among the four serotypes, individual contexts have variation between serotypes. By examining the nucleotide composition images of codon pairs generated from Anaconda analysis (data not shown), it was found that (A)(A/T)(A)-(A)(A/T)(A) sequences are the most abundant codon contexts in the DENV genome. Conversely, the (C/G)(C/A)(C/G)-(C/G)(C/A)(C/G) patterns are generally avoided in the codon context sequences. Based on frequencies of individual codon contexts among the four serotypes, the Anaconda algorithm was also used to group the serotypes, which revealed that codon context patterns of DENV-1 and DENV-3 are more closely related than DENV-1 vs. DENV-2 or DENV-1 vs. DENV-4 (data not shown). DENV-2 and DENV-3 are closer in the codon context patterns than that of DENV-2 vs. DENV-4 or DENV-1 vs. DENV-2.

### Identification of sites under selection

The DENV isolates were further characterized to identify sites within codons under positive and negative selection within each serotype. Using fixed effects likelihood methods (see Methods), we identified 521-743 sites within serotypes that are associated with negative selection in DENV (Additional file 6). However, the sites under position selection in the DENV genome were exceptionally low (less than 4) in each serotype. The majority of the selected sites are localized in the NS3 and NS5 genes (Table 4). The sequences encoding the 2k signal peptide [33] of NS4A and also sequences of anchored capsid protein C show the least number of selected sites suggesting extensive bias in natural selection of individual genes of DENV. Many of the negatively selected sites show fixation tendency within serotypes. A total of 287 of the 743 negatively selected sites (38.6%) of DENV-1, 165 of the 693 negatively selected sites (23.8%) of DENV-2, and 190 of the 521 negatively selected sites (36.4%) of DENV-3 showed fixation tendency where frequency of each site was > 95% in one geographical region compared to < 5% frequency in the other (i.e. Asian and American populations). In DENV-4, a total of 33 of the 615 negatively selected sites (5.3%) showed similar fixation tendency either in the South American population or the Central American population. None of positively selected sites, however, show such fixation tendency within any serotype. These results suggest that although selected sites are generally thought to be beneficial for the organism, the negatively selected sites rather than the positively selected sites seem to be beneficial to DENV.

**Table 4:** Number of sites selected in the investigated samples of the four dengue virus serotypes

**Gene**	**DENV-1**	**DENV-2**	**DENV-3**	**DENV-4**
2K protein	1	6	4	3
anchored capsid protein C	9	11	11	13
nonstructural protein NS2B	29	27	20	27
membrane glycoprotein precursor M	35	33	24	32
nonstructural protein NS4A	36	23	24	31
nonstructural protein NS2A	49	55	28	35
nonstructural protein NS4B	51	57	36	46
nonstructural protein NS1	82	77	53	56
envelope protein E	114	82	85	89
nonstructural protein NS3	138	140	109	124
nonstructural protein NS5	199	182	127	159

### Relationship between intracodon recombination and purifying selection in dengue virus

**Figure 4:**
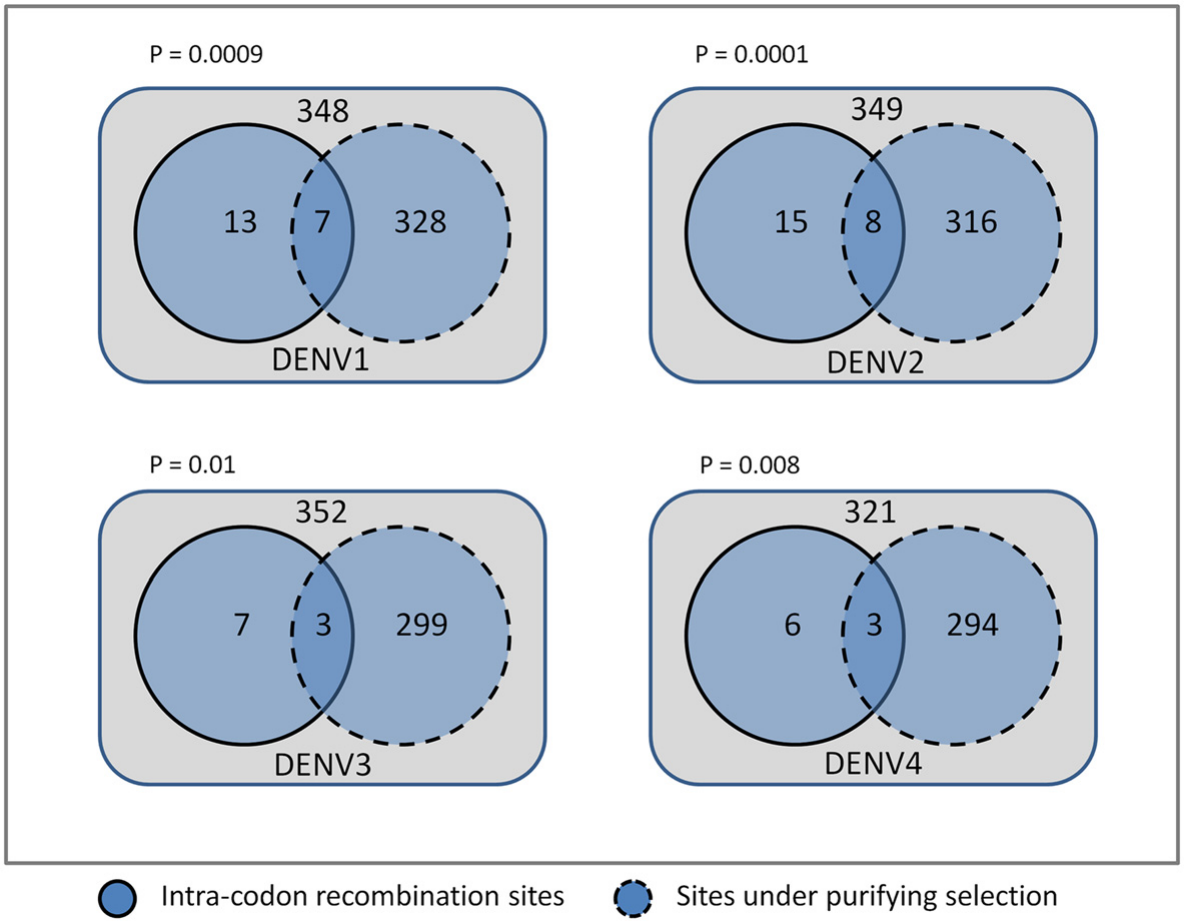
**Distribution of purifying selection sites and sites of intracodon recombination.** Enrichment of sites under purifying selection among codon sites showing evidence of intracodon recombination in dengue virus serotypes. The total number of sites that show evidence of recombination is shown within the box but outside the two circles. Of these, the number of sites showing intracodon recombination and sites under purifying selection is shown in solid and dashed line circles. The number of sites under both selection and intracodon recombination is shown in the overlapping region of the two circles. The p-value above each serotype (shown as DENV-1, DENV-2, DENV-3 and DENV-4) represents the statistical significance of a hypergeometric test to reject the null assumption that the observed enrichment pattern is a random event.

To test whether recombination may have an effect on the purifying sites in the DENV genome, we determined the population recombination events within each serotype using the ‘PAIRWISE’ composite likelihood method described by McVean *et al*. (2002) [28]. The sites with minimum numbers of recombination events in samples were determined from ‘PAIRWISE’ analysis according to Hudson and Kaplan (1985) [29]. Figure 4 shows the hypergeometric distribution of the recombination sites between sites associated with intracodon recombination and sites under purifying selection within each serotype. Of all the sites showing evidence of recombination (ranging from 321 to 352 among the serotypes), the majority of them (more than 84%) corresponded to sites under purifying selection in the four serotypes. The analysis further shows that intracodon recombination does occur in the DENV genome, albeit with low frequency (< 5% of all recombination sites) within the four serotypes. The number of intracodon recombination sites ranges from 9 to 23 as shown in Figure 4. Although intracodon recombination is low in frequency, as much as 50% of the pairwise sites are under purifying selection. This clearly suggests significant enrichment (p < 0.05) of sites under purifying selection with the sites associated with intracodon recombination in DENV.

**Figure 5:**
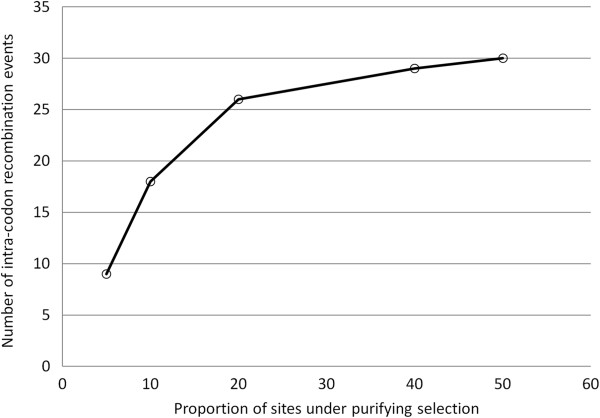
**Relationship between purifying selection and intracodon recombination.** The x-axis shows the proportion of sites under purifying selection and the y-axis shows the number of intracodon recombination events in the simulated sequences.

To investigate whether this pattern of intracodon recombination is a true representation of the DENV genome, we performed coalescent simulation of codon sequences to infer the relationship between intracodon recombination and purifying selection in simulated sequences. The simulations were performed according to methods of Arenas and Posada (2010) [20]. Five independent simulations were performed with the same parameters, but with increasing proportions of sites (5%, 10%, 20%, 40% and 50%) with omega = 0.1. The omega value 0.1 was selected based on observed average dN/dS values of DENV sequences. The results of simulation data clearly showed that with the increase in the proportion of purifying selection, the number of intracodon recombination events increases, but to a certain limit (n = 26). Then the number of intracodon recombination events decreases even if the sites under purifying selection increase in number (Figure 5). This suggests that enrichment of purified selection sites among the sites associated with intracodon recombination is not a random chance of observation in the sampled sequences but may be a real representation of association between the two factors. However, there may be a threshold for the cause/effect of purified selection on numbers of intracodon recombination events in DENV as suggested by the simulation results.

## Discussion

The present investigation was carried out to better understand the molecular evolution of coding sequences of DENV isolates of the four serotypes from different geographical regions. The study utilized a random sampling of sequence data from the GRID project, which is intended to provide a detailed description of DENV ecology and evolution across time and space among a collection of world-wide isolates. Our efforts were limited to enhancing our understanding of polymorphisms in codon sequences and how these relate to recombination and selection sites in DENV.

The phylogenetic relationships among DENV genomes corresponded to their geographical origins, indicating phylogeographic diversity of gene sequences among isolates. The mean distance of genetic diversity within serotypes varies according to the extent of geographical dispersal of isolates. Serotype 4 isolates, which were limited to Central and South American origin, showed relatively low genetic diversity compared to serotypes 1, 2 or 3 that consisted of isolates from countries in both Asia and the Americas. Although we have focused on intra-serotype genetic diversity in this work, comparisons between serotypes of DENV isolates has also been reported by other studies [34,35]. According to these studies, it is believed that clade replacement and related stochastic events associated with geographical structures may lead to serotype differentiation. However, the substitution rates are very homogenous across serotypes [34].

Results from our study showed that positively selected sites are exceptionally rare in DENV isolates of each serotype. Moreover, no sites of positive selection were found in DENV isolates from the Americas (serotype 4). It has been estimated that more than 90% of all non-synonymous mutations in the DENV genome lack any evidence of benefit for the organism and can be considered deleterious [36]. In that study, Holmes found that non-synonymous variations are abundant in DENV populations within individual humans, whereas the frequency of non-synonymous mutations in inter-host comparisons is very low. Thus, the loss of long-term non-synonymous variation is the signature of extensive purifying selection in the DENV genome.

We asked whether fixation of specific synonymous codons between American and Asian DENV is associated with selection for codon optimization within serotypes. To determine that, the synonymous mutations that resulted in generation of preferred and non-preferred codons were counted in both populations, and our results show that synonymous substitutions between Asian and American DENV isolates are significantly associated with codon preferences or non-preferences. One of the significant observations from this study is that several codons undergo fixed substitutions (Additional file 2) at the 3^rd^ position (mostly A to G changes) between Asian and American DENV isolates. These silent substitutions show extensive changes in the RSCU value of the codons. In many cases, the RSCU is less than 0.5 in one geographic population but greater than 2 in the other geographic population, suggesting that they are used in a very biased manner between Asian or American DENV isolates. Codon usage bias is an important evolutionary feature of the DENV genome, where it has been suggested that closely related isolates have more similar codon usage patterns than more distantly related isolates [37]. The same study [37] further showed that codon bias can be used as an indicator of serotype differentiation in DENV. In this context, our results suggest that fixed mutations at silent positions of codons contribute to biased usage of codons between geographical samples of dengue virus. This further indicates that substitutions, even if they are silent, can play an important role in geographical diversity in the virus. Whether fixation of such sites is associated with evolutionary benefit to the virus is yet to be investigated, although it is possible that codon bias can be beneficial [38]. The relevance of codon bias of DENV is also thought to a co-evolutionary relationship with the vector mosquito *Aedes aegypti*[39]. In this context, it has been shown that codon bias of genes is the most influential factor among other intrinsic features of mosquito genes to have a significant effect on transcriptional responsiveness to infection by DENV [40]. Thus, it seems likely that fixed changes between Asian and American DENV isolates pertaining to differential usage of synonymous codons may have a role in molecular interaction with the mosquito genotypes prevailing in the regions [41-43].

Rate of mutation in arthropod-borne viruses (arboviruses) is generally lower compared to that of viruses that infect vertebrate hosts directly [44]. The trade-off effect on arboviruses including DENV obligated to adapt alternatively into the invertebrate vector and vertebrate host is believed to be associated with reduced rate of mutations. Thus, DENV evolution is also subjected to trade-off effects by the vector wherein fitness of the virus improves when it replicates in one cell line compared to alternative passages in both mosquito and human cells [45]. It has been suggested that the trade-off effect may be responsible for evolution of distinct lineages within DENV serotype as seen in the case of serotype 1 in Columbia [46]. According to this study [46], hyperendemic infections of dengue in humans contributed to relaxing the trade-off effect on the virus from the mosquito vector population in the region. Although elevated mutational rate in viruses is primarily due to the lack of proof-reading activity of RNA-dependent RNA-polymerases, relaxation of vector associated trade-off effects on virus may also lead to increased rate of substitutions in dengue virus [46]. Based on these studies and the studies suggesting that nucleotide substitution patterns may have co-evolutionary links between mosquito and virus [39], it is thus likely that evolution of dengue virus is intricately dependent upon selective pressure resulting from both host (relating to immune status) and mosquito (relating to vectorial capacity) [47]. Thus, spatial population and phylogenetic analyses of DENV are essential for better understanding the history and epidemiology of the disease [48].

According to the selection-mutation-drift theory [49], some codons are used preferentially over alternate synonymous codons for better efficiency of translation of a gene, while mutation and drift balances the selection force on that gene. In this context, the results from our investigation indicated an excess of non-preferred codons over preferred codons suggesting that synonymous sites are under relaxed selection in DENV. Thus, the balance between selection and mutation likely contributes to the widespread prevalence of silent sites which are weakly selected in the DENV genome. While GC percentage can have a significant influence on codon bias, the DENV genome shows ~ 50% GC content in the coding sequences, wherein the effective number of codons within each serotype typically varies from 48 to 51. At the same time, it is known that changes in the 1^st^ and 2^nd^ positions can have an effect on compositional bias of amino acids of proteins in insects [50-52]. In the DENV genome, we found that the fixed mutations leading to differential usage of codons are primarily associated with four specific amino acids: Gly, Pro, Ser and Thr. Although the importance of fixed mutations for these specific amino acids in DENV is not clear, it has been shown that either specific residues in the glycoprotein or motifs of Gly-, Pro-, Ser- and Thr-rich regions are likely to have important roles in host infectivity probably by protein-protein interaction or simply protein function for attachment with host glycoproteins [53-55].

In the DENV genome, a majority of the pair-wise recombination sites correspond to sites with synonymous substitutions. However, recombination was also evident between sites with non-synonymous substitutions. Depending upon whether both the sites in the pair-wise recombination are either synonymous or non-synonymous, there exists a significant relationship between synonymous/ non-synonymous sites and sites with inter- and intracodon recombination (data not shown). This shows that while recombination between non-synonymous sites represents nearly similar numbers of inter- and intracodon sites, recombination events between synonymous sites are significantly biased towards inter-codon recombination. The inter-codon recombination events in the DENV genome occur primarily between the 3^rd^ position of two codons whereas the intracodon recombination events occur among all the three codon positions without any bias. The 3^rd^ codon position being the silent substitution position, recombination between silent sites of codons explains higher synonymous changes than non-synonymous changes (purifying selection) throughout the DENV genome. The results of our study further reveal that the frequency of intracodon recombination has a significant association with the extent of purifying selection in DENV (Figure 4). This suggests that intracodon recombination contributes to relatively higher synonymous than non-synonymous changes per site in DENV. It is likely that intracodon recombination may be responsible in part for a reduction in non-synonymous mutations of DENV among human hosts. Non-synonymous variations are abundant in viral populations within individual humans, whereas the frequency of non-synonymous substitutions in inter-host comparisons is very low [36].

Our data has further revealed that only specific residues of the DENV polyprotein are associated with intracodon recombination where substitutions occur at multiple positions within codons (data not shown). These codons primarily encode leucine, and to some extent serine and arginine, and are often associated with synonymous substitutions in the 1^st^ as well as the 3^rd^ position. Moreover, the results from simulation studies (Figure 5) indicate that the relationship between intracodon recombination and purifying selection is non-linear, and also has a threshold point after which we may not observe more intracodon recombination even if the number of sites under purifying selection increases.

## Conclusions

The results obtained from this study provide insights into the nature of nucleotide substitution patterns in DENV serotypes in a genome-wide manner and reveal evidence for translational selection of specific sites between Asian and American DENV isolates. Specific sites in the DENV genome are associated with biased usages of synonymous codons between Asian and American DENV populations suggesting that translational selection has a role in the evolution of dengue virus. The study also indicates that fixation of specific mutations leads to codon usage bias in dengue virus. One of the interesting findings is that only three amino acids (Leu, Ser and Arg) in the DENV polyprotein are associated with multiple substitutions within codons. Furthermore, the results of this study suggest, for the first time, that intracodon recombination does occur in DENV and is significantly associated with the extent of purifying selection in each serotype. This suggests that genetic recombination within codons plays an important role in maintaining extensive purifying selection of DENV in natural populations.

## Abbreviations

DENV: Dengue virus;Leu: Leucine;Ser: Serine;Arg: Arginine;Pro: Proline;A: Adenine;C: Cytosine;G: Guanine;T: Thymine;RSCU: Relative Synonymous Codon Usage

## Competing interests

The authors declare that they have no competing interests.

## Authors’ contributions

Conceived and designed the experiments: SKB. Analyzed the data: SKB. Contributed reagents/materials/analysis tools: SKB, DWS. Wrote the paper: SKB, DWS. Agree with the manuscript's results and conclusions: SKB, DWS. Both authors read and approved the final manuscript.

## Authors’ information

SKB’s current work focuses on genetic and genomic dissection of dengue susceptibility of *Aedes aegypti* vector mosquitoes. He has a broad interest in vector borne diseases with emphasis on vector-virus interactions, disease ecology and evolution and vector competence of disease transmission. He works as a Research Assistant Professor in the Department of Biological Sciences and the Eck Institute for Global Health at the University of Notre Dame, Indiana. DWS’s research is broadly focused on mosquito genetics and genomics. His work primarily concerns genetic analysis of mosquito vector competence to various pathogens as well as on development and application of molecular tools to investigate population biology of mosquitoes. He is a Professor of Biological Sciences and the Director of the Eck Institute for Global Health at the University of Notre Dame, Indiana.

## Supplementary Material

Additional file 1: Table S1 List of GenBank accession numbers of dengue virus samples investigated in the study. The country and year of collection of samples are also provided. Click here for file

Additional file 2: Table S2 Relative rate of nucleotide substitutions (based on HKY85 model) within serotypes of dengue virus. Click here for file

Additional file 3: Table S3 Distribution of synonymous (syn) and non-synonymous (non-syn) sites among different genes of dengue virus. The numbers in parenthesis are counts of substitutions that are fixed within serotypes. The p value shows statistical significance of association between synonymous or nonsynonymous sites with or without tendency of fixation in each gene. Click here for file

Additional file 4 Codons that are used more often than expected in one sample (RSCU >2 *) but less than expected (RSCU < 0.5) in the other in the same serotype of dengue virus. Click here for file

Additional file 5: Figure S1 Condon context patterns of DENV 1, 2, 3 and 4. Click here for file

Additional file 6 List of positively and negatively selected sites in dengue virus genes. Click here for file
