# Supplementary material for: Nucleotide substitutions in dengue virus serotypes from Asian and American countries: insights into intracodon recombination and purifying selection
Source: BMC Microbiol. 2013 Feb 14;13:37. doi: 10.1186/1471-2180-13-37 (PMC3598932; doi:10.1186/1471-2180-13-37)
Supplement: Additional file 2: Table S2 — Relative rate of nucleotide substitutions (based on HKY85 model) within serotypes of dengue virus. [file 1471-2180-13-37-S2.docx]

Table S2. Relative rate of nucleotide substitutions (based on HKY85 model) within serotypes of dengue virus.

| DENV-1_Relative Substitution Rate | | | |  |
| --- | --- | --- | --- | --- |
|  | A | C | G | T |
| A | * | 0.058163 | 1 | 0.058163 |
| C | - | * | 0.058163 | 1 |
| G | - | - | * | 0.058163 |
| T | - | - | - | * |
| DENV-2_Relative Substitution Rate | | | |  |
|  | A | C | G | T |
| A | * | 0.048417 | 1 | 0.048417 |
| C | - | * | 0.048417 | 1 |
| G | - | - | * | 0.048417 |
| T | - | - | - | * |
| DENV-3_Relative Substitution Rate | | | |  |
|  | A | C | G | T |
| A | * | 0.057228 | 1 | 0.057228 |
| C | - | * | 0.057228 | 1 |
| G | - | - | * | 0.057228 |
| T | - | - | - | * |
| DENV-4_Relative Substitution Rate | | | |  |
|  | A | C | G | T |
| A | * | 0.047487 | 1 | 0.047487 |
| C | - | * | 0.047487 | 1 |
| G | - | - | * | 0.047487 |
| T | - | - | - | * |
