# Supplementary material for: Nucleotide substitutions in dengue virus serotypes from Asian and American countries: insights into intracodon recombination and purifying selection
Source: BMC Microbiol. 2013 Feb 14;13:37. doi: 10.1186/1471-2180-13-37 (PMC3598932; doi:10.1186/1471-2180-13-37)
Supplement: Additional file 3: Table S3 — Distribution of synonymous (syn) and non-synonymous (non-syn) sites among different genes of dengue virus. The numbers in parenthesis are counts of substitutions that are fixed within serotypes. The p value shows statistical significance of association between synonymous or nonsynonymous sites with or without tendency of fixation in each gene. [file 1471-2180-13-37-S3.docx]

Supplementary Table 3. Distribution of synonymous (syn) and non-synonymous (non-syn) sites among different genes of dengue virus. The numbers in parenthesis are counts of substitutions that are fixed within serotypes. The p value shows statistical significance of association between synonymous or nonsynonymous sites with or without tendency of fixation in each gene.

| **DENV1** |  |  | p value | Significant |
| --- | --- | --- | --- | --- |
| Gene | Total syn sites (fixed syn sites) | Total non-syn sites (fixed non-syn sites) | | |
| anchored capsid protein C | 35 (15) | 16 (3) | 0.122287 |  |
| membrane glycoprotein precursor M | 72 (31) | 19 (3) | 0.03423052 | * |
| envelope protein E | 234 (86) | 62 (14) | 0.04890274 | * |
| nonstructural protein NS1 | 150 (55) | 53 (14) | 0.2374193 |  |
| nonstructural protein NS2A | 98 (37) | 57 (10) | 0.01080502 | * |
| nonstructural protein NS2B | 54 (27) | 17 (5) | 0.169188 |  |
| nonstructural protein NS3 | 286 (104) | 35 (15) | 0.4632591 |  |
| nonstructural protein NS4A | 60 (23) | 9 (3) | 1 |  |
| 2K protein | 9 (5) | 8 (3) | 0.6371863 |  |
| nonstructural protein NS4B | 117 (37) | 11 (2) | 0.5018676 |  |
| nonstructural protein NS5 | 369 (136) | 75 (9) | 1.18362E-05 | * |
| **DENV2** |  |  |  |  |
| Gene | Total syn sites (fixed syn sites) | Total non-syn sites (fixed non-syn sites) | | |
| anchored capsid protein C | 43 (11) | 12 (7) | 0.04335832 | * |
| membrane glycoprotein precursor M | 63 (21) | 15 (3) | 0.3703917 |  |
| envelope protein E | 175 (53) | 62 (14) | 0.3246842 |  |
| nonstructural protein NS1 | 139 (38) | 31 (12) | 0.2750958 |  |
| nonstructural protein NS2A | 104 (30) | 35 (10) | 1 |  |
| nonstructural protein NS2B | 48 (18) | 14 (4) | 0.7523731 |  |
| nonstructural protein NS3 | 253 (73) | 38 (9) | 0.5673676 |  |
| nonstructural protein NS4A | 50 (11) | 6 (2) | 0.6145865 |  |
| 2K protein | 11 (3) | 6 (4) | 0.1617647 |  |
| nonstructural protein NS4B | 95 (30) | 25 (1) | 0.004042603 | * |
| nonstructural protein NS5 | 346 (100) | 69 (28) | 0.06366546 |  |
| **DENV3** |  |  |  |  |
| Gene | Total syn sites (fixed syn sites) | Total non-syn sites (fixed non-syn sites) | | |
| anchored capsid protein C | 33 (10) | 14 (4) | 1 |  |
| membrane glycoprotein precursor M | 54 (19) | 14 (3) | 0.5226077 |  |
| envelope protein E | 191 (76) | 53 (16) | 0.2620761 |  |
| nonstructural protein NS1 | 129 (39) | 33 (6) | 0.1966303 |  |
| nonstructural protein NS2A | 93 (43) | 25 (8) | 0.2576205 |  |
| nonstructural protein NS2B | 44 (15) | 8 (1) | 0.4088664 |  |
| nonstructural protein NS3 | 239 (95) | 42 (6) | 0.001455558 | * |
| nonstructural protein NS4A | 51 (16) | 16 (6) | 0.7621386 |  |
| 2K protein | 8 (3) | 8 (0) | 0.2 |  |
| nonstructural protein NS4B | 97 (38) | 13 (5) | 1 |  |
| nonstructural protein NS5 | 308 (110) | 90 (23) | 0.07654251 |  |
| **DENV4** |  |  |  |  |
| Gene | Total syn sites (fixed syn sites) | Total non-syn sites (fixed non-syn sites) | | |
| anchored capsid protein C | 40 (0) | 9 (0) | 1 |  |
| membrane glycoprotein precursor M | 75 (1) | 15 (0) | 1 |  |
| envelope protein E | 185 (8) | 67 (3) | 1 |  |
| nonstructural protein NS1 | 142 (7) | 45 (3) | 0.7056191 |  |
| nonstructural protein NS2A | 82 (3) | 30 (0) | 0.5628729 |  |
| nonstructural protein NS2B | 52 (3) | 16 (0) | 1 |  |
| nonstructural protein NS3 | 262 (11) | 32 (1) | 1 |  |
| nonstructural protein NS4A | 58 (0) | 13 (0) | 1 |  |
| 2K protein | 11 (0) | 3 (0) | 1 |  |
| nonstructural protein NS4B | 97 (9) | 19 (0) | 0.3516722 |  |
| nonstructural protein NS5 | 364 (14) | 62 (0) | 0.2379893 |  |
