# Supplementary material for: Nucleotide substitutions in dengue virus serotypes from Asian and American countries: insights into intracodon recombination and purifying selection
Source: BMC Microbiol. 2013 Feb 14;13:37. doi: 10.1186/1471-2180-13-37 (PMC3598932; doi:10.1186/1471-2180-13-37)
Supplement: Additional file 5: Figure S1 — Condon context patterns of DENV 1, 2, 3 and 4. [file 1471-2180-13-37-S5.docx]

Supplementary Figure 1. Condon context patterns of DENV 1, 2, 3 and 4.
